# (*E*)-5-(2-Thienylmethyl­eneamino)quinolin-8-ol

**DOI:** 10.1107/S1600536807066652

**Published:** 2007-12-21

**Authors:** Stéphane Dufresne, Alex N. Bourque, W. G. Skene

**Affiliations:** a Department of Chemistry, University of Montreal, CP 6128, succ. Centre-ville, Montréal, Québec, Canada H3C 3J7

## Abstract

Two mol­ecules of the title compound, C_14_H_10_N_2_OS, are hydrogen bonded about a center of inversion. In the mol­ecule, the two aromatic rings are twisted by 37.27 (5)° with respect to one another. The azomethine bond is in the *E* configuration.

## Related literature

For information about the utility of azomethines, see: Dufresne *et al.* (2006 ▶); Skene & Dufresne (2006 ▶). For related structures, see: Chen *et al.* (1999 ▶). For an analog with an aryl ring in place of the thienyl ring, see Manecke *et al.* (1972 ▶).
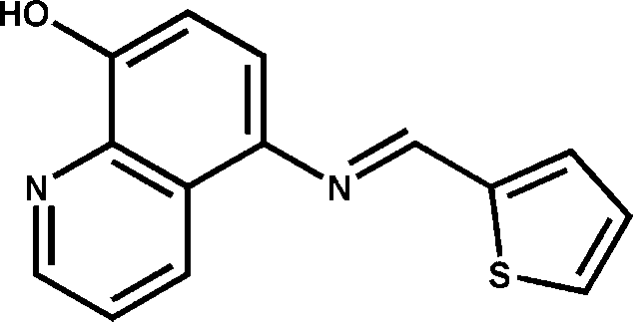


## Experimental

### 

#### Crystal data

C_14_H_10_N_2_OS
*M* *_r_* = 254.30
Monoclinic, 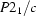

*a* = 7.6798 (4) Å
*b* = 9.8592 (4) Å
*c* = 15.7512 (7) Å
β = 92.926 (2)°
*V* = 1191.07 (9) Å^3^
*Z* = 4
Cu *K*α radiation
μ = 2.31 mm^−1^
*T* = 150 (2) K
0.07 × 0.05 × 0.05 mm

#### Data collection

Bruker SMART 6K diffractometer
Absorption correction: multi-scan (*SADABS*; Sheldrick, 1996 ▶) *T*_min_ = 0.855, *T*_max_ = 0.893
31904 measured reflections
2377 independent reflections
2152 reflections with *I* > 2σ(*I*)
*R*_int_ = 0.064

#### Refinement

*R*[*F* ^2^ > 2σ(*F* ^2^)] = 0.054
*wR*(*F* ^2^) = 0.139
*S* = 1.11
2377 reflections
164 parameters
H-atom parameters constrained
Δρ_max_ = 0.42 e Å^−3^
Δρ_min_ = −0.62 e Å^−3^

### 

Data collection: *SMART* (Bruker, 2003 ▶); cell refinement: *SAINT* (Bruker, 2004 ▶); data reduction: *SAINT*; program(s) used to solve structure: *SHELXS97* (Sheldrick, 1997 ▶); program(s) used to refine structure: *SHELXL97* (Sheldrick, 1997 ▶); molecular graphics: *ORTEP-3* (Farrugia, 1997 ▶) and *SHELXTL* (Bruker, 1997 ▶); software used to prepare material for publication: *UdMX* (Marris, 2004 ▶).

## Supplementary Material

Crystal structure: contains datablocks I, global. DOI: 10.1107/S1600536807066652/ng2406sup1.cif

Structure factors: contains datablocks I. DOI: 10.1107/S1600536807066652/ng2406Isup2.hkl

Additional supplementary materials:  crystallographic information; 3D view; checkCIF report

## Figures and Tables

**Table 1:** Hydrogen-bond geometry (Å, °)

*D*—H⋯*A*	*D*—H	H⋯*A*	*D*⋯*A*	*D*—H⋯*A*
O1—H1⋯N1^i^	0.84	2.27	2.927 (2)	136

Symmetry code: (i) 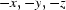.

**Fig. 1.**
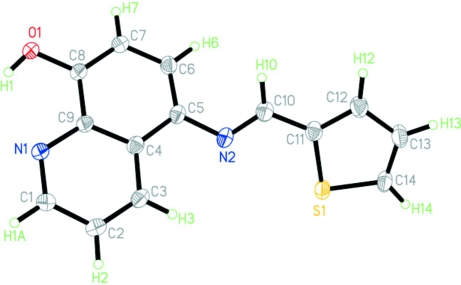
ORTEP representation of (I) with the numbering scheme adopted (Farrugia 1997). Ellipsoids drawn at 30% probability level.

**Fig. 2.**
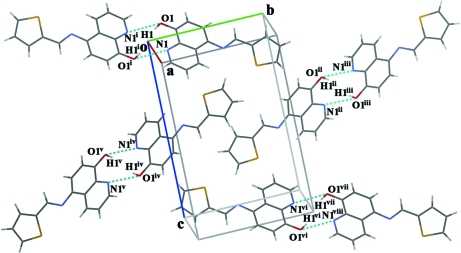
The lattice structure of (I) showing hydrogen bonding. [Symmetry codes: (i) -x, -y, -z; (ii)

## supplementary crystallographic information

### Comment

Compound (I) was prepared as a new ligand for metal-ligand charge transfer complexes. The structure of (I) consists of quinolin-2-ol covalently linked to a thiophene unit by an azomethine bond with more stable E isomer being observed. The crystal structure has a P21/c symmetry as seen in figure 2. No solvent molecules or counter-ions were found in the crystal structure.

The bond lengths and angles of the quinolin-2-ol moiety are within 0.013 Å and 1°, respectively, to comparable structures (Chen *et al.*, 1999). The bond lengths of the azomethine bond for C5—N2, N2—C10 and C10—C11 are 1.421 (2), 1.276 (2) and 1.446 (2) Å, respectively. The bond lengths are comparable to an all thiophene azomethine analogue (Dufresne *et al.*, 2006) whose analogues bond lengths are 1.388 (3), 1.272 (3) and 1.441 (4) Å, respectively.

The mean planes of the two aryl moieties are twisted by 37.27 (5)° from the azomethine bond to which they are connected. This angle is smaller, *i.e.* 65°, (Manecke *et al.*, 1972) than its homoaryl analogue. Steric hindrance between H6 and H10 is responsible for the twist between the mean planes similar to a thiophene azomethine, whose aryl mean planes are twisted by 33° Skene *et al.*, 2006).

Hydrogen bonding takes place between two quinolin-8-ol moieties to form a supramolecular dimer. Figure 2 shows the two symmetry related hydrogen bonds between O1—H1···N1î^ and O1î^-H1î^···N1 that form the dimer. The length and the angle of this bond are 2.927 (2) Å and 136°, respectively. The two quinolin-2-ol involved in the hydrogen bonding are shifted by 0.593 Å.

### Experimental

The title compound was synthesized by means of an acid catalyzed condensation of 5-amino-8-hydroxyquinoline with 2-thiophenecarboxaldehyde in ethanol with catalytic trifluoroacetic acid. The reaction was held at reflux for 20 h with stirring, cooled to room temperature and the volume reduced. Ice-cold distilled water was added to this solution causing a yellow solid to precipitate. The yellow solid was collected, washed with water and then dried under reduced pressure overnight. Crystals were obtained by slow evaporation of a concentrated solution of (1) in acetone.

### Refinement

H atoms were placed in calculated positions (C—H = 0.95 Å) and included in the refinement in the riding-model approximation, with *U*_iso_(H) = 1.2 *U*_eq_(C). The hydrogen on the hydroxyl group was placed in calculated position (O—H = 0.84 Å, C—O—H = 109.5°) and included in the refinement in the riding-model approximation with *U*_iso_(H) = 1.5 *U*_eq_(O).

### Crystal data

**Table:**

C_14_H_10_N_2_OS	*F*_000_ = 528
*M**_r_* = 254.30	*D*_x_ = 1.418 Mg m^−^^3^
Monoclinic, *P*2_1_/*c*	Cu *K*α radiation λ = 1.54178 Å
Hall symbol: -P 2ybc	Cell parameters from 7426 reflections
*a* = 7.6798 (4) Å	θ = 5.3–72.9º
*b* = 9.8592 (4) Å	µ = 2.31 mm^−^^1^
*c* = 15.7512 (7) Å	*T* = 150 (2) K
β = 92.926 (2)º	Block, yellow
*V* = 1191.07 (9) Å^3^	0.07 × 0.05 × 0.05 mm
*Z* = 4	

### Data collection

**Table:**

Bruker SMART 6000 diffractometer	2377 independent reflections
Radiation source: Rotating Anode	2152 reflections with *I* > 2σ(*I*)
Monochromator: Montel 200 optics	*R*_int_ = 0.064
Detector resolution: 5.5 pixels mm^-1^	θ_max_ = 73.2º
*T* = 150(2) K	θ_min_ = 5.3º
φ and ω scans	*h* = −9→9
Absorption correction: multi-scan(SADABS; Sheldrick, 1996)	*k* = −12→11
*T*_min_ = 0.855, *T*_max_ = 0.893	*l* = −19→19
31904 measured reflections	

### Refinement

**Table:**

Refinement on *F*^2^	Secondary atom site location: difference Fourier map
Least-squares matrix: full	Hydrogen site location: inferred from neighbouring sites
*R*[*F*^2^ > 2σ(*F*^2^)] = 0.054	H-atom parameters constrained
*wR*(*F*^2^) = 0.139	*w* = 1/[σ^2^(*F*_o_^2^) + (0.0968*P*)^2^ + 0.157*P*] where *P* = (*F*_o_^2^ + 2*F*_c_^2^)/3
*S* = 1.11	(Δ/σ)_max_ < 0.001
2377 reflections	Δρ_max_ = 0.42 e Å^−^^3^
164 parameters	Δρ_min_ = −0.62 e Å^−^^3^
Primary atom site location: structure-invariant direct methods	Extinction correction: none

### Special details

**Table:**

Geometry. All e.s.d.'s (except the e.s.d. in the dihedral angle between two l.s. planes) are estimated using the full covariance matrix. The cell e.s.d.'s are taken into account individually in the estimation of e.s.d.'s in distances, angles and torsion angles; correlations between e.s.d.'s in cell parameters are only used when they are defined by crystal symmetry. An approximate (isotropic) treatment of cell e.s.d.'s is used for estimating e.s.d.'s involving l.s. planes.
Refinement. Refinement of *F*^2^ against ALL reflections. The weighted *R*-factor *wR* and goodness of fit *S* are based on *F*^2^, conventional *R*-factors *R* are based on *F*, with *F* set to zero for negative *F*^2^. The threshold expression of *F*^2^ > σ(*F*^2^) is used only for calculating *R*-factors(gt) *etc*. and is not relevant to the choice of reflections for refinement. *R*-factors based on *F*^2^ are statistically about twice as large as those based on *F*, and *R*- factors based on ALL data will be even larger.

### Fractional atomic coordinates and isotropic or equivalent isotropic displacement parameters (Å^2^)

**Table:**

	*x*	*y*	*z*	*U*_iso_*/*U*_eq_	
S1	0.43366 (6)	0.82399 (4)	0.21499 (3)	0.0464 (2)	
O1	0.10263 (18)	0.13156 (13)	−0.09352 (7)	0.0446 (3)	
H1	0.0556	0.0746	−0.0623	0.067*	
N1	0.0099 (2)	0.14942 (15)	0.07194 (9)	0.0394 (3)	
N2	0.29207 (18)	0.59227 (14)	0.09825 (9)	0.0406 (3)	
C1	−0.0336 (3)	0.16030 (18)	0.15190 (11)	0.0449 (4)	
H1A	−0.0985	0.0886	0.1752	0.054*	
C2	0.0107 (3)	0.2718 (2)	0.20431 (11)	0.0460 (4)	
H2	−0.0225	0.2741	0.2616	0.055*	
C3	0.1018 (2)	0.37649 (19)	0.17187 (11)	0.0419 (4)	
H3	0.1327	0.4524	0.2066	0.050*	
C4	0.1505 (2)	0.37229 (17)	0.08639 (10)	0.0361 (4)	
C5	0.2413 (2)	0.47908 (17)	0.04695 (11)	0.0373 (4)	
C6	0.2810 (2)	0.46462 (17)	−0.03706 (11)	0.0393 (4)	
H6	0.3422	0.5352	−0.0637	0.047*	
C7	0.2332 (2)	0.34810 (17)	−0.08420 (11)	0.0397 (4)	
H7	0.2618	0.3414	−0.1420	0.048*	
C8	0.1456 (2)	0.24404 (17)	−0.04735 (10)	0.0367 (4)	
C9	0.1013 (2)	0.25436 (16)	0.03912 (9)	0.0354 (4)	
C10	0.3002 (2)	0.70894 (18)	0.06355 (12)	0.0424 (4)	
H10	0.2655	0.7176	0.0050	0.051*	
C11	0.3600 (2)	0.82854 (16)	0.10947 (12)	0.0420 (4)	
C12	0.3671 (3)	0.95730 (19)	0.07858 (13)	0.0529 (5)	
H12	0.3312	0.9809	0.0219	0.063*	
C13	0.4333 (3)	1.0526 (2)	0.13929 (14)	0.0554 (5)	
H13	0.4470	1.1465	0.1277	0.066*	
C14	0.4751 (3)	0.99469 (18)	0.21584 (12)	0.0474 (4)	
H14	0.5216	1.0430	0.2640	0.057*	

### Atomic displacement parameters (Å^2^)

**Table:**

	*U*^11^	*U*^22^	*U*^33^	*U*^12^	*U*^13^	*U*^23^
S1	0.0614 (3)	0.0305 (3)	0.0465 (3)	0.00053 (17)	−0.0045 (2)	−0.00223 (15)
O1	0.0632 (8)	0.0393 (7)	0.0312 (6)	−0.0135 (6)	0.0022 (5)	−0.0040 (5)
N1	0.0495 (8)	0.0376 (7)	0.0305 (7)	−0.0066 (6)	−0.0034 (6)	0.0002 (5)
N2	0.0402 (7)	0.0350 (8)	0.0461 (8)	−0.0009 (6)	−0.0026 (6)	−0.0081 (6)
C1	0.0552 (10)	0.0457 (10)	0.0337 (9)	−0.0069 (8)	0.0013 (7)	0.0017 (7)
C2	0.0556 (10)	0.0502 (10)	0.0321 (8)	−0.0020 (8)	0.0006 (7)	−0.0043 (7)
C3	0.0462 (9)	0.0418 (9)	0.0371 (8)	0.0000 (7)	−0.0033 (7)	−0.0080 (7)
C4	0.0356 (8)	0.0356 (8)	0.0365 (8)	0.0022 (7)	−0.0050 (6)	−0.0033 (6)
C5	0.0358 (8)	0.0332 (8)	0.0424 (8)	0.0004 (6)	−0.0040 (6)	−0.0045 (6)
C6	0.0392 (8)	0.0358 (9)	0.0427 (9)	−0.0050 (6)	0.0006 (6)	0.0001 (6)
C7	0.0443 (9)	0.0401 (9)	0.0347 (8)	−0.0039 (7)	0.0011 (7)	−0.0018 (7)
C8	0.0406 (8)	0.0365 (8)	0.0326 (8)	−0.0032 (7)	−0.0034 (6)	−0.0032 (6)
C9	0.0371 (8)	0.0361 (8)	0.0325 (8)	−0.0022 (6)	−0.0046 (6)	−0.0004 (6)
C10	0.0407 (9)	0.0380 (9)	0.0477 (10)	0.0032 (7)	−0.0068 (7)	−0.0028 (7)
C11	0.0409 (9)	0.0358 (9)	0.0485 (10)	0.0036 (7)	−0.0051 (7)	−0.0027 (7)
C12	0.0643 (12)	0.0380 (10)	0.0547 (11)	0.0023 (9)	−0.0136 (9)	0.0024 (8)
C13	0.0691 (13)	0.0313 (9)	0.0639 (12)	0.0002 (8)	−0.0130 (10)	0.0005 (8)
C14	0.0541 (10)	0.0317 (9)	0.0555 (11)	0.0014 (8)	−0.0064 (8)	−0.0072 (7)

### Geometric parameters (Å, °)

**Table:**

S1—C14	1.7127 (18)	C4—C5	1.423 (2)
S1—C11	1.7289 (19)	C5—C6	1.380 (2)
O1—C8	1.358 (2)	C6—C7	1.406 (2)
O1—H1	0.8400	C6—H6	0.9500
N1—C1	1.324 (2)	C7—C8	1.372 (2)
N1—C9	1.367 (2)	C7—H7	0.9500
N2—C10	1.276 (2)	C8—C9	1.424 (2)
N2—C5	1.421 (2)	C10—C11	1.446 (2)
C1—C2	1.406 (3)	C10—H10	0.9500
C1—H1A	0.9500	C11—C12	1.362 (3)
C2—C3	1.361 (3)	C12—C13	1.417 (3)
C2—H2	0.9500	C12—H12	0.9500
C3—C4	1.416 (2)	C13—C14	1.358 (3)
C3—H3	0.9500	C13—H13	0.9500
C4—C9	1.421 (2)	C14—H14	0.9500
			
C14—S1—C11	91.92 (9)	C8—C7—H7	119.7
C8—O1—H1	109.5	C6—C7—H7	119.7
C1—N1—C9	117.25 (14)	O1—C8—C7	119.70 (14)
C10—N2—C5	118.84 (15)	O1—C8—C9	120.47 (14)
N1—C1—C2	123.84 (17)	C7—C8—C9	119.83 (15)
N1—C1—H1A	118.1	N1—C9—C4	123.30 (14)
C2—C1—H1A	118.1	N1—C9—C8	117.39 (14)
C3—C2—C1	119.08 (16)	C4—C9—C8	119.30 (15)
C3—C2—H2	120.5	N2—C10—C11	122.82 (17)
C1—C2—H2	120.5	N2—C10—H10	118.6
C2—C3—C4	120.04 (16)	C11—C10—H10	118.6
C2—C3—H3	120.0	C12—C11—C10	126.81 (18)
C4—C3—H3	120.0	C12—C11—S1	110.52 (14)
C3—C4—C9	116.48 (15)	C10—C11—S1	122.67 (13)
C3—C4—C5	123.54 (15)	C11—C12—C13	113.39 (18)
C9—C4—C5	119.98 (15)	C11—C12—H12	123.3
C6—C5—N2	123.97 (15)	C13—C12—H12	123.3
C6—C5—C4	118.63 (15)	C14—C13—C12	112.38 (18)
N2—C5—C4	117.35 (15)	C14—C13—H13	123.8
C5—C6—C7	121.74 (16)	C12—C13—H13	123.8
C5—C6—H6	119.1	C13—C14—S1	111.78 (14)
C7—C6—H6	119.1	C13—C14—H14	124.1
C8—C7—C6	120.53 (16)	S1—C14—H14	124.1
			
C9—N1—C1—C2	−0.8 (3)	C3—C4—C9—N1	0.8 (2)
N1—C1—C2—C3	0.8 (3)	C5—C4—C9—N1	−178.15 (15)
C1—C2—C3—C4	0.1 (3)	C3—C4—C9—C8	179.70 (15)
C2—C3—C4—C9	−0.8 (2)	C5—C4—C9—C8	0.7 (2)
C2—C3—C4—C5	178.12 (16)	O1—C8—C9—N1	−2.1 (2)
C10—N2—C5—C6	33.6 (2)	C7—C8—C9—N1	178.23 (15)
C10—N2—C5—C4	−149.15 (16)	O1—C8—C9—C4	178.95 (14)
C3—C4—C5—C6	−179.47 (15)	C7—C8—C9—C4	−0.7 (2)
C9—C4—C5—C6	−0.6 (2)	C5—N2—C10—C11	−176.40 (16)
C3—C4—C5—N2	3.2 (2)	N2—C10—C11—C12	−177.5 (2)
C9—C4—C5—N2	−177.93 (14)	N2—C10—C11—S1	2.4 (3)
N2—C5—C6—C7	177.58 (15)	C14—S1—C11—C12	−0.57 (17)
C4—C5—C6—C7	0.4 (2)	C14—S1—C11—C10	179.52 (17)
C5—C6—C7—C8	−0.4 (3)	C10—C11—C12—C13	−179.54 (19)
C6—C7—C8—O1	−179.12 (15)	S1—C11—C12—C13	0.6 (2)
C6—C7—C8—C9	0.5 (3)	C11—C12—C13—C14	−0.2 (3)
C1—N1—C9—C4	0.0 (2)	C12—C13—C14—S1	−0.2 (2)
C1—N1—C9—C8	−178.93 (16)	C11—S1—C14—C13	0.45 (17)

### Hydrogen-bond geometry (Å, °)

**Table:**

*D*—H···*A*	*D*—H	H···*A*	*D*···*A*	*D*—H···*A*
O1—H1···N1^i^	0.84	2.27	2.927 (2)	136

Symmetry codes: (i) −*x*, −*y*, −*z*.
